# Influence of Filler in the Form of Waste Wood Flour and Microcellulose on the Mechanical, Thermal, and Morphological Characteristics of Hierarchical Epoxy Composites

**DOI:** 10.3390/molecules31020363

**Published:** 2026-01-20

**Authors:** Anna Sienkiewicz, Piotr Czub

**Affiliations:** Department of Chemistry and Technology of Polymers, Faculty of Chemical Engineering and Technology, Cracow University of Technology, Warszawska Str. 24, 31-155 Kraków, Poland; piotr.czub@pk.edu.pl

**Keywords:** biopolymers, epoxy resin, wood filler, microcellulose, epoxy composites, chemical modification

## Abstract

In response to growing interest in green additives derived from natural raw materials or post-production waste of natural origin, epoxy compositions containing the additive in the form of waste wood flour and microcellulose were prepared. The research involved the chemical modification of the additive through a two-stage silanization process using 3-aminopropyltriethoxysilane. Followed by filler’s characterization using Fourier Transformed Infrared Spectroscopy (FT-IR) to analyze the modification in chemical structure, Wide Angle X-Ray Diffraction (WAXD) to detect differences in crystal structure, and Scanning Electron Microscopy (SEM) to observe morphological changes. Next, waste oak flour (WF) and microcrystalline cellulose (MCC) were used in unmodified and silanized form (sil-WF and sil-MCC, respectively) to prepare epoxy composites, followed by testing their influence on the mechanical (hardness, tensile strength, flexural strength, compressive strength, and impact strength), thermal, and morphological characteristics of epoxy composites based on Epidian 6. Comparing the effect of modification on the properties of the analyzed additives, it was found that silanization had a larger impact on increasing the interaction of the waste wood flour with the epoxy matrix than silanization of MCC due to a lesser tendency of the sil-WF than the sil-MCC to agglomerate. An enhanced interaction of sil-WF with the polymer resulted in improved mechanical properties. Composite EP/sil-WF (cured epoxy composite based on low-molecular-weight epoxy resin Epidian 6 filled with 5 wt.% of silanized wood flour) was characterized by improved flexural (61.97 MPa) and compressive properties (69.1 MPa) compared to both EP/WF (cured epoxy composite based on low-molecular-weight epoxy resin Epidian 6 filled with 5 wt.% of unmodified wood flour) (42.39 MPa and 61.0 MPa) and the unfilled reference composition (54.55 MPa and 67.4 MPa, respectively). Moreover, compositions containing a cellulosic additive were characterized by better impact properties than the reference composition.

## 1. Introduction

Considering functional properties such as higher strength and modulus, specific deformation behavior, lightweight nature, and good corrosion resistance, fiber-reinforced polymer composites have garnered wider interest compared to monolithic polymer materials. For several years, fibers, such as carbon, glass, and aramid, were the most popular additives due to their superior physical and mechanical properties. However, over the past few years, an increase in pro-ecological activities has been observed. It is, in this case, understood as reducing the actions aimed at limiting the use of depleting fossil fuel deposits, limiting the production of greenhouse gases, contributing to global warming, and striving to obtain materials that will not remain in landfills for years after they become unusable and that also potentially decompose faster [1,2]. In this trend, raw materials of natural origin have received more attention worldwide. Fiber-reinforcing hybrid composites are a large group of these materials. The application of natural fibers is determined by technological aspects, aiming to obtain materials with better, comparable, or new properties that are competitive with materials commonly available on the market. The ecological aspect of obtaining materials using natural raw materials is also important, and it is becoming increasingly significant nowadays. To reduce plastic waste, governments around the world are introducing significant pro-ecological activities consisting of promoting the segregation and recycling of used plastic materials, encouraging the production of materials with a high share of raw materials of natural origin, obtaining biodegradable polymer materials, or even further enacting laws prohibiting the production of non-biodegradable plastics. The implementation of ecological regulations also includes the production of epoxy resins. Actions are being taken towards reducing the use of hazardous fossil-based materials to bio-based alternatives (plant oils and lignin) [3,4], reducing VOCs by adopting sustainable practices [5,6], improving end-of-life management for recyclability [7,8], and complying with the strict European Union’s directives like the Green Deal [9]. Currently, epoxy resins are obtained mostly using petrochemical monomers. However, intensified research is being carried out to obtain more ecological epoxy materials. Studies include the application of bio-based monomers, including lignin-based phenols, vanillin, cardanol, isosorbide, and terpene derivatives as a replacement for bisphenol A [10,11,12,13]; epoxidized vegetable oils in place of low/medium-molar-mass resins, as well as hydroxylated oils in place of bisphenols in the epoxy fusion process with Bisphenol A (BPA) or BPA-based epoxy resin [14], or bio-based curing agents [15,16]. Next to the application of natural raw materials in the process of the synthesis of epoxy resins, more eco-friendly epoxy materials might be obtained in the form of composite materials using waste residues of natural origin, including vegetable fibers and other lignocellulosic particles such as nutshell, coconut shell, etc. The introduction of waste bio-based materials in conventional polymer materials is one way to improve the bio-content of these materials, but also to some extent reduce the cost of their production.

A significant part of the research on the use of raw materials of natural origin includes research on the use of cellulosic raw materials. Attempts to use cellulose-based raw materials are associated with their wide availability, low cost and density, relatively large ratio of surface to volume, as well as reactive surfaces, biodegradability, and good mechanical properties [17]. The main components of natural fibers are cellulose, hemicellulose, and lignin. Cellulose is a linear polymer of glucose (β-1,4-linked D-glucose units); its content varies from 40 to 60% of the fiber depending on the type and part of the plant. Cellulose is characterized by a crystalline structure, with the highest content of hydroxyl groups (18.5 mmol/g). Among other lignocellulosic components, it is considered to have the best mechanical properties and provides structural strength and rigidity to plant cell walls [18]. Hemicellulose is a branched polymer of various sugars (e.g., xylose, mannose, glucose, and galactose) that binds cellulose fibers together. Lignin is an irregular aromatic polymer that consists of phenylpropanoid units.

Lignocellulosic additives, mostly often used as natural reinforcing materials for polymeric composites, are wood fillers obtained from different tree species, jute, hemp, flax fibers, cotton, sisal fibers, bagasse fibers, coir, wheat, or rapeseed straw. Fillers, applied as fibers of different sizes and dimensions, are obtained from plants directly or as one of the methods of managing waste products from plant processing [19]. It is important to remember that the final performance of composites depends on the ratio and individual properties of the applied fibers and polymer matrix, the size and orientation of fibers [20]. However, compared to the conventional synthetic fibers, natural reinforcement additives are characterized by relatively poor mechanical properties [21]. Improving the performance properties of natural fiber composites is possible by modifying the additive through changing chemical composition of fibers (throughout chemical modification, such as alkali treatment [22], silane treatment [23], acetylation [24], etherification [25], or peroxide tratment [26]) or just by changing the surface and structural properties of fibers without modification of its chemical composition (via physical modifications, such as cold plasma treatment [27] or electric discharge [28]). It is also worth highlighting that combining different physical and chemical modifications has also been investigated.

Recently, microcrystalline cellulose (MCC) has found special interest among fillers of natural origin. Microcellulose is a promising cellulosic additive for polymer composite materials, as it significantly enhances the mechanical, morphological, and thermal properties of the composites. Alpha cellulose crystalline phases, which are arranged at a nanometer scale, create a microcellulose structure [29]. Moreover, microcrystalline cellulose is characterized by a higher specific surface area (smaller diameter) than natural fibers, leading to enhanced interactions and bonding possibilities with the polymer matrix [30]. Sosiati et al. [31] obtained MCC/sisal/PMMA composites for dental applications, characterized by hardness, flexural strength, and impact strength that can compete with dental composite materials such as TiO_2_ nanoparticle/PMMA, ZrO_2_/glass fiber/PMMA, and cellulose/PMMA. The addition of MCC into the polyester matrix of ramie/polyester laminated composites increases the tensile strength and elastic modulus of the obtained material by 18% and 21%, respectively [32]. Microcellulose dispersion within the poly(butylene adipate-co-terephthalate) matrix film [33], which could potentially be used as a biodegradable film in food packaging applications, results in delayed degradation of the material, leading to improved thermal stability. It also makes changes within the mechanical properties, increasing its stiffness while decreasing tensile strength. It is worth highlighting that microcellulose, like other lignocellulosic reinforcement fillers, is subjected to surface functionalization. Modifications with, e.g., stearic and oleic acids, ethylenediamine, poly(methylhydro)siloxane, silane coupling, and pyridone diester, improve the microcellulose performance due to enhanced adhesion abilities. Yuan et al. [34] modified MCC with phytic acid by phosphorylation reaction and obtained a promising flame-retardant material characterized by low heat-release performance and good char-forming ability during thermal degradation. The introduction of silane-modified microcellulose to the epoxy composition by Wang et al. [35] led strengthening effect of silanized-MCC, better interfacial compatibility between the filler and polymer matrix, which influenced better dispersity of MCC in the composition, and the improvement of fire performance of the composite by the formation of a better char layer.

The manuscript aims to study the influence of cellulosic fillers of natural origin in pure and silanized form on the properties of fiber-reinforced epoxy resin composites. Within the research, we performed silanization of fillers with 3-aminopropyltriethoxysilane. Having regard to the results of our previous studies, we attempt to improve the properties of epoxy composites containing natural additives by introducing a synergistic effect of fillers. The fiber-reinforced epoxy composites were obtained using fillers in their natural and silanized form, such as the waste oak flour from the processing of parquet flooring, microcrystalline cellulose, and a combination of cellulosic fillers. The authors obtained composites containing 5 wt.% of an additive of natural origin using the dispersion of additives in bisphenol A epoxy resin cured with isophorone–diamine. The presented research discusses the influence of modification on the chemical structure and morphology of the filler particles, as well as the properties of composites containing one or two different fillers.

## 2. Results and Discussion

### 2.1. Silanization of Filler

Wood flour (WF) and microcrystalline cellulose (MCC) were used as fillers for the epoxy resins. The WF filler was obtained as waste from the sanding process of oak parquet floors. To increase the compatibility of the filler with the hydrophobic epoxy matrix, each filler was chemically modified by combining two methods used for natural fillers: mercerization and silanization.

**Figure 1:**
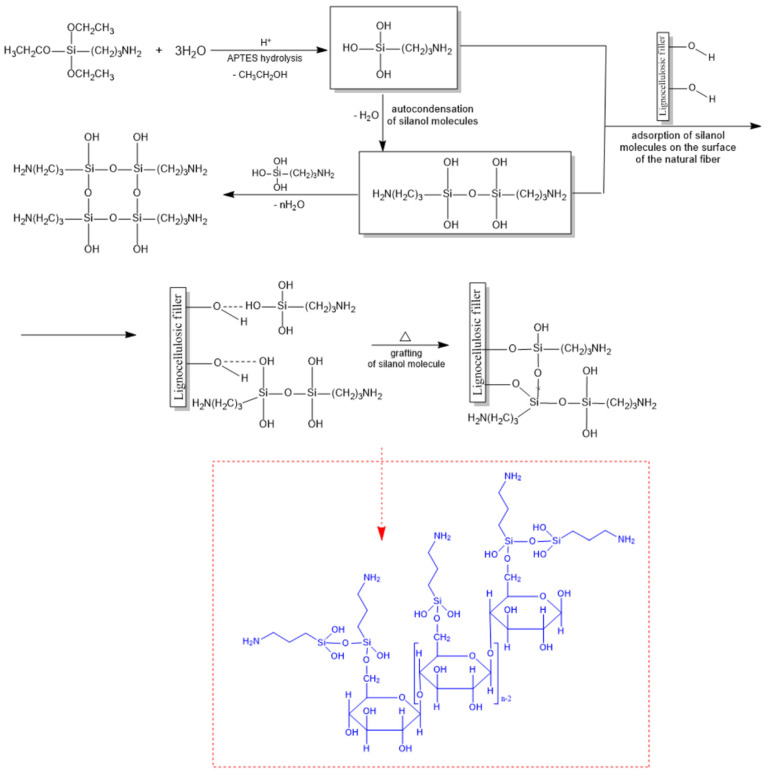
Scheme of grafting silanol on lignocellulosic filler during the silanization process.

Mercerization of natural fibers is a process that involves subjecting them to an alkaline treatment, most often with a sodium hydroxide solution. This method of modifying natural fibers results in the partial removal of compounds covering their surface, such as hemicellulose and lignin, while increasing their roughness. The alkaline solution also causes strong swelling, leading to a loosening of the cellulose structure [36]. Silanization, in turn, is a chemical modification method used for lignocellulosic additives and is based on the use of a chemical compound from the silane group (e.g., 3-aminopropyltriethoxysilane (APTES), γ-aminopropyltriethoxysilane (APS), or γ-diethylenetriaminepropyltrimethoxysilane (TAS)). These types of coupling substances create a bridge between the additive and the polymer matrix, increasing their mutual compatibility (Figure 1).

The non-reactive alkyl group of the silane, due to its nonpolar nature, can increase the affinity of the natural filler with the nonpolar polymer matrix. At the appropriate pH, silane hydrolyzes in the presence of water. This reaction results in the formation of alkoxysilanols and alcohols. The silane groups condense to form a siloxane. This is caused by the presence of hydrogen bonds between silane groups, leading to the formation of siloxane bridges. When siloxanes come into contact with the surface of natural fibers, they substitute hydrogen atoms for hydroxyl groups.

The mercerization process of the dried filler was carried out using a 10% aqueous solution of sodium hydroxide (NaOH) at 25 °C (please see Section 3.1.1 and Section 3.1.2). The wet waste was then silanized using a 3-aminopropyltriethoxysilane solution in a water–methanol system (1:1) with a pH of 4. The desired pH was achieved by gradually adding glacial acetic acid in portions to a previously prepared methanol/water solution. Each filler was modified by silanization for 20 h at room temperature. As a result of the modification, both fillers, wood waste and microcrystalline cellulose, swelled, resulting in difficulties during filtration. A color change from yellow–brown to dark brown was also observed. In the next step, distilled water was added to the resulting waste, thoroughly mixed with a mechanical stirrer, and filtered again. The obtained modified wood waste was dried in an oven at 60 °C for 24 h.

**Figure 2:**
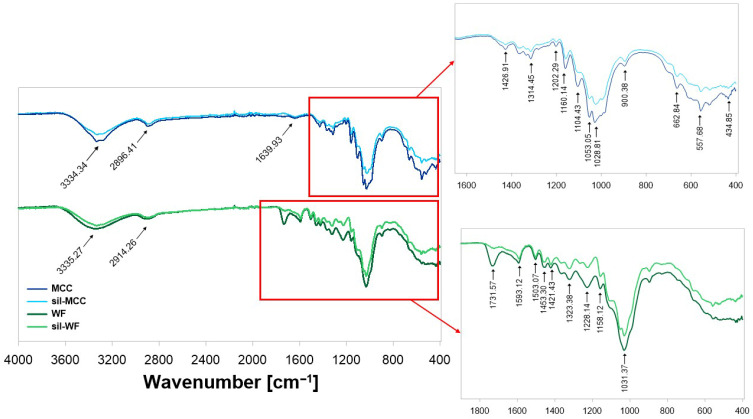
FT-IR spectrum of fillers for epoxy composites in the form of unmodified (WF) and silanized (sil-WF) wood flour and unmodified (MCC) and silanized microcrystalline cellulose (sil-MCC).

In the next stage of the study, the chemical structure of fillers in the form of unmodified (WF) and silanized (sil-WF) wood flour and unmodified (MCC) and silanized microcrystalline cellulose (sil-MCC) was analyzed (Figure 2 and Table 1). The recorded FT-IR spectra were presented as the dependence of transmittance T [%] on the wavenumber value v [cm^−1^]. The FT-IR interpretation of the observed changes related to the modification of the cellulose additive is in accordance with all our recent studies [37,38,39,40] and includes procedures regarding performing the analysis in the same manner, maintaining the same measurement conditions, and an interpretation procedure for qualitative comparison of the recorded spectra.

The results of FT-IR analysis, presented above, for untreated MCC and WF show typical bands, which are characteristic of this kind of raw material [45,46]. There is a broad band in the range of 3637–3000 cm^−1^ for microcrystalline cellulose and 3600–3000 cm^−1^ for oak waste flour due to the presence of hydroxyl groups in cellulose (-OH, stretching vibrations in OH groups H-bond). The next band recorded in the spectrum, at 3000–2800 cm^−1^, which is characterized by a relatively small intensity, may come from the -C-H stretching vibrations in CH_2_, -CH_2_, and -CH groups of cellulose or hemicellulose, which constitute wood flour. Meanwhile, bands around 1740 cm^−1^ and 1600 cm^−1^ indicate the presence of the C=O stretching vibration of carbonyl, carboxyl, and acetyl groups. The band around 1640 cm^−1^ is related to the C=O stretching vibrations of aromatic ketones, as with the one at 1654 cm^−1^ (-OH bending vibration). The signal recorded at 1415 cm^−1^ comes from the -CH_2_ symmetric bending vibration. In turn, the band at 1360 cm^−1^ corresponds to the deformation vibrations of the -C-H bending vibration. The band in the range of 1350–1260 cm^−1^ is assigned to the -C-O stretching vibrations. In turn, bands at 1223 cm^−1^ and at 1083 cm^−1^ are most likely characteristic of -C-O-C- stretching vibrations. The -C-OH stretching vibrations of lignin were recorded in the spectrum at 1026 cm^−1^.

Comparing the recorded FT-IR spectra of MCC and sil-MCC, a reduction in the intensity of most vibrations in the sil-MCC spectrum can be observed. It is primarily visible in the characteristic stretching vibrations of the -OH groups with measurements of 3600–3000 cm^−1^. Additionally, the H-O-H bending vibration of absorbed moisture detected around 1640 cm^−1^ in the MCC spectrum also decreased after the silanization process. This indicates a reduction in the hydrophilic character of the modified microcrystalline cellulose due to the chemical modification. Moreover, in the spectrum of sil-MCC, similarly to Neves et al.’s [47] research, the characteristic vibrations for Si-O at 1080 cm^−1^ were observed for microcellulose after the silanization process.

Comparing the FT-IR spectra of modified (WF) and silanized (sil-WF) wood flour, the following differences can be observed. The first is the presence of a characteristic band of Si-O stretching vibrations at 1540 cm^−1^. Its presence confirms the modification of waste wood flour using APTES. Another significant difference is the absence of a band at 1732 cm^−1^ in the sil-WF spectrum, which appears in the spectrum of unmodified WF. This band characterizes C=O vibrations originating from hemicellulose or lignin. Their absence may indicate partial removal of hemicellulose, which occurred as a result of the first stage of waste modification, i.e., the mercerization of wood flour. Zhang et al. [48] reached similar conclusions when analyzing a sample of bamboo fibers using FT-IR. Additionally, an increase in the intensity of the band in the 1150–1050 cm^−1^ range was observed in the sil-WF spectrum, associated with the overlap of vibrations corresponding to the presence of hydroxyl and ether groups, as well as Si-O vibrations. Similar effects were observed after silanization of plant waste in the form of corn leaves by Liu et al. [49]. They also observed the appearance of a signal characteristic of Si-O vibrations in the spectra of silane-modified fibers.

**Figure 3:**
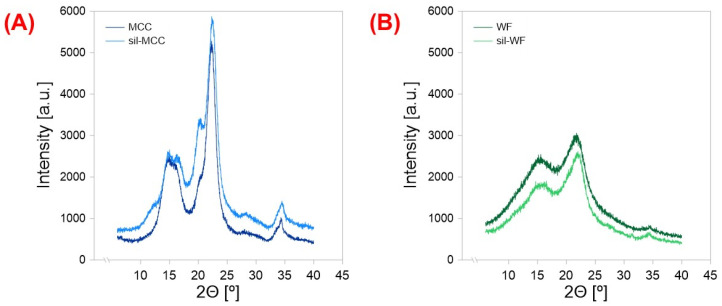
XRD rentgenograms of microcellulose and silanized MCC (**A**) and untreated waste wood flour and silanized WF (**B**).

The combination of silanization with mercerization was dictated by the facilitation of the silanization process. It Is known that during mercerization, the crystalline structure of cellulose tends to relax [50]. Such a transformation in the case of typical lignocellulosic raw fibers results in partial decrystallization, leading to an increase in the average content of the amorphous regions, which typically are more prone to chemical penetration than the crystalline regions [51]. To analyze the crystalline structure of unmodified and modified filler samples, XRD analysis was performed (Figure 3). In the case of both modified additives, no significant changes related to the appearance of supplementary crystallographic planes were found. For MCC and sil-MCC (Figure 3A), only cellulose I characteristic peaks at 14.8°, 16.2°, 22.5°, and 34.3° were found [52]. For the samples of wood flour and sil-WF, these specific peaks were also noted, but in the case of waste wood flour, the intensities of peaks, especially those at 14.9°, 21.9°, and 34.4°, were much lower than in the case of microcrystalline cellulose.

**Figure 4:**
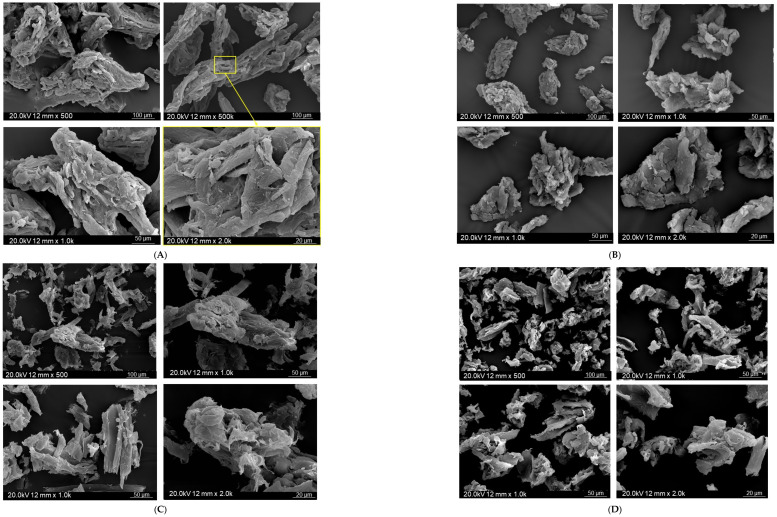
SEM micrographs of additive particles before and after silanization: microcrystalline cellulose (**A**), silanized microcellulose (**B**), waste wood flour (**C**) [40], and silanized waste wood flour (**D**).

**Figure 5:**
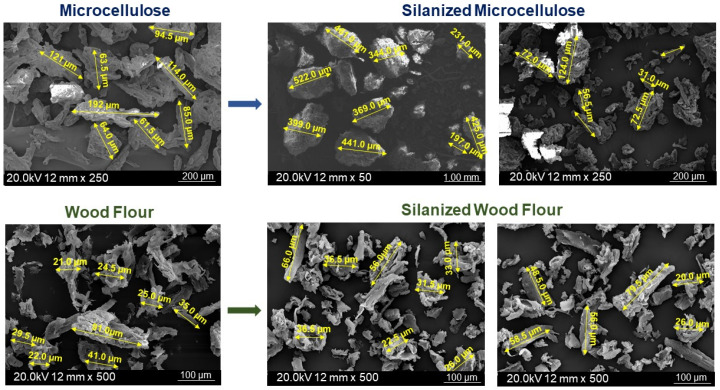
SEM micrographs of additive particles before and after silanization–the influence of chemical modification on particle size [40].

Figure 4 and Figure 5 present the SEM microimages of the additive particles before and after the silanization. It should be highlighted that the comparison of the morphology and the grain sizes of additives was made upon the detailed observation of the entire sample subjected to the SEM analysis. The most representative microimages for each cellulosic additive are presented below.

Regardless of the type of additive, its particles create irregular structures of various shapes and sizes. They are in the form of bundles with various irregular cross-interlocking junctions. The structure of the microcellulose surface before silanization (Figure 4A) is smooth and rather compact, with a clearly defined rod-like layer. This compact structure is probably a result of the presence of three-dimensional internal bonds of crystalline structure, which were isolated during the production process of microcrystalline cellulose from raw lignocellulosic materials [46]. Comparing the morphology of MCC and WF (Figure 4A,C), a relatively more ordered structure with layered small rods in MCC microphotographs might indicate the removal of lignin, hemicellulose, and other impurities during the isolation procedure. The surface of waste wood flour presented in the SEM microphotographs (Figure 4C) was rougher than that of MCC, with numerous irregular thin strips protruding above the surface that probably resulted from the earlier wood processing. As mentioned earlier, based on the analysis of the FT-IR spectrum, the performed chemical modification via mercerization, combined with silanization of the biofiller, had an impact on its chemical structure. SEM microphotographs also show changes in the morphological appearance of the additive particles. Due to the modification, an enlargement of particles was observed. Here, it should be highlighted that comparision of the average particle sizes in the individual micrographs and the made observations regarding the morphology of the individual additives and the influence of the conducted chemical modification were conducted with the respect that in the case of all additives, MCC, sil-MCC, WF, and sil- WF, particle sizes may result from various grains’ structures oriented with respect to each other differently. Such a situation may be particularly characteristic of MCC and sil-MCC due to the already mentioned specific compact rod-like structure. Due to the slightly less compact structure of WF particles pointed out phenomenon might occur to a lesser extent. In connection with the above, the most visible changes were observed in the case of the silanized microcellulose with a predominance of agglomerates of particles with sizes of approximately 200–550 µm (Figure 5), while the dimensions of particles of unmodified MCC were within the range 60–200 µm. For the unmodified wood samples, most of the wood particles were about 23–39 μm, with a single larger particle. After the silanization, slight changes in the wood filler particle sizes were observed. Silanized wood particle sizes were in the range of 20–80 μm. In the case of sil-MCC and sil-WF, it was found that fillers were fragmented, and the surface of the fillers became rougher, implying the success of the performed modification. Additionally, in the case of sil-MCC, the irregular rod-like structure is still present, but it is less distinct, and the grains become even more compact locally, creating agglomerations.

### 2.2. Preparation of Epoxy Composites

**Figure 6:**
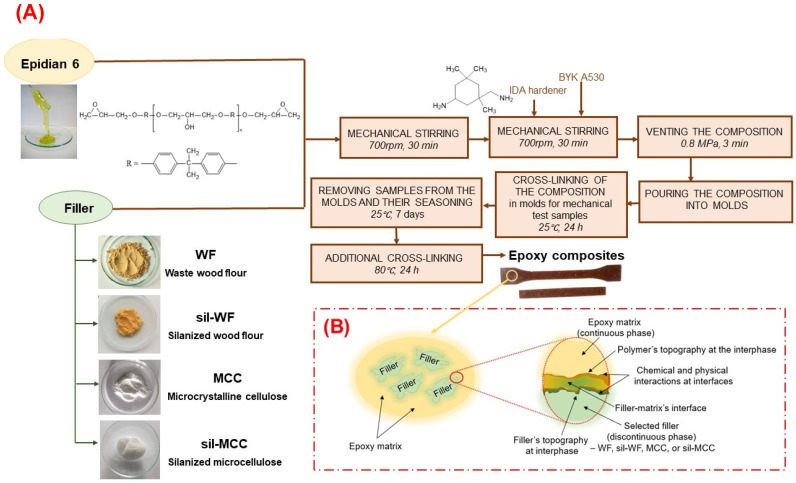
Scheme of the preparation of epoxy composites (**A**) with the schematic presentation of the distribution of the additive in the polymer matrix, along with probable interaction of polymer composite components (**B**).

In the next stage of the research, compositions based on the low-molecular-weight epoxy resin Epidian 6 were prepared. Seven compositions were prepared (Figure 6, Table 2), including a reference composition (EP), compositions with one filler in an amount of 5 wt.% of waste wood flour (EP/WF and EP/sil-WF) or 1 wt.% of microcrystalline cellulose (EP/MCC and EP/sil-MCC), and compositions containing two fillers with a total additive content of 5% by weight (4 wt.% of silanized wood flour and 1 wt.% of unmodified or silanized microcellulose–EP/sil-WF/MCC and EP/sil-MD/sil-MCC compositions, respectively).

**Figure 7:**
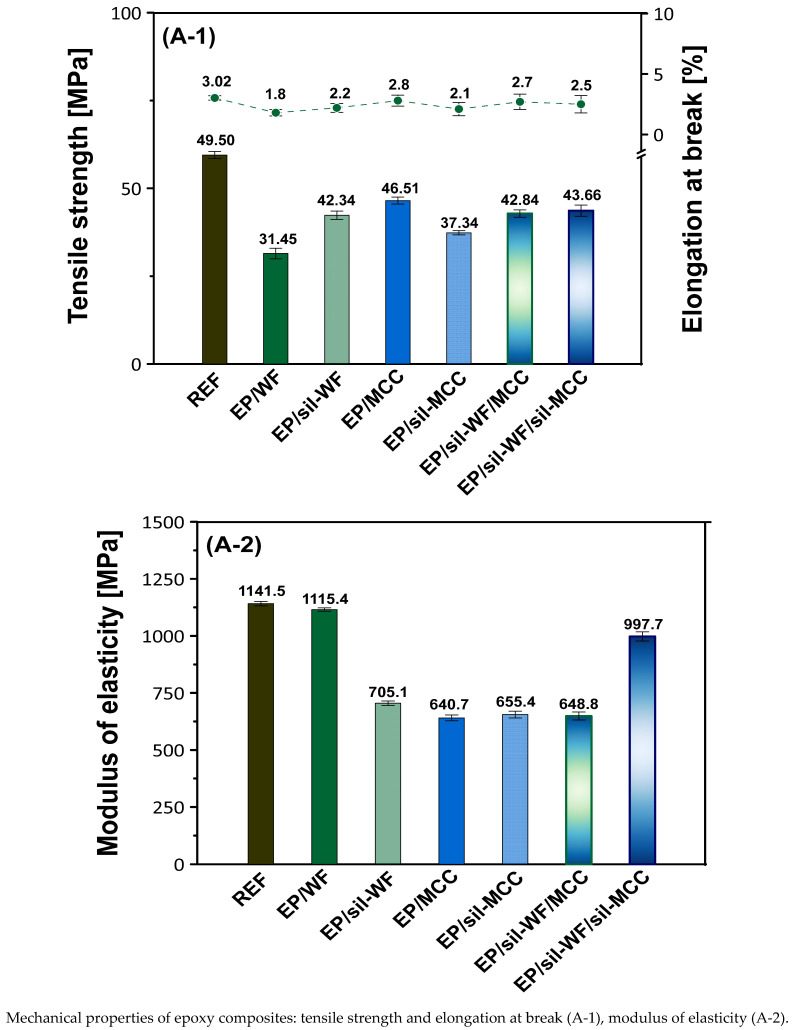

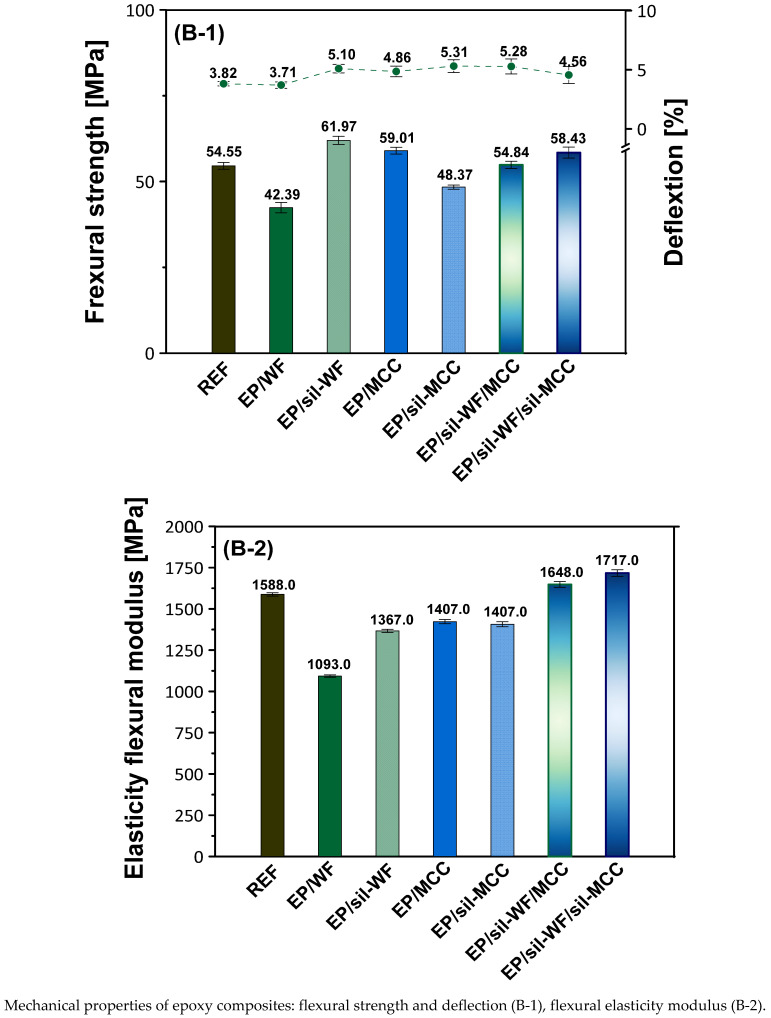

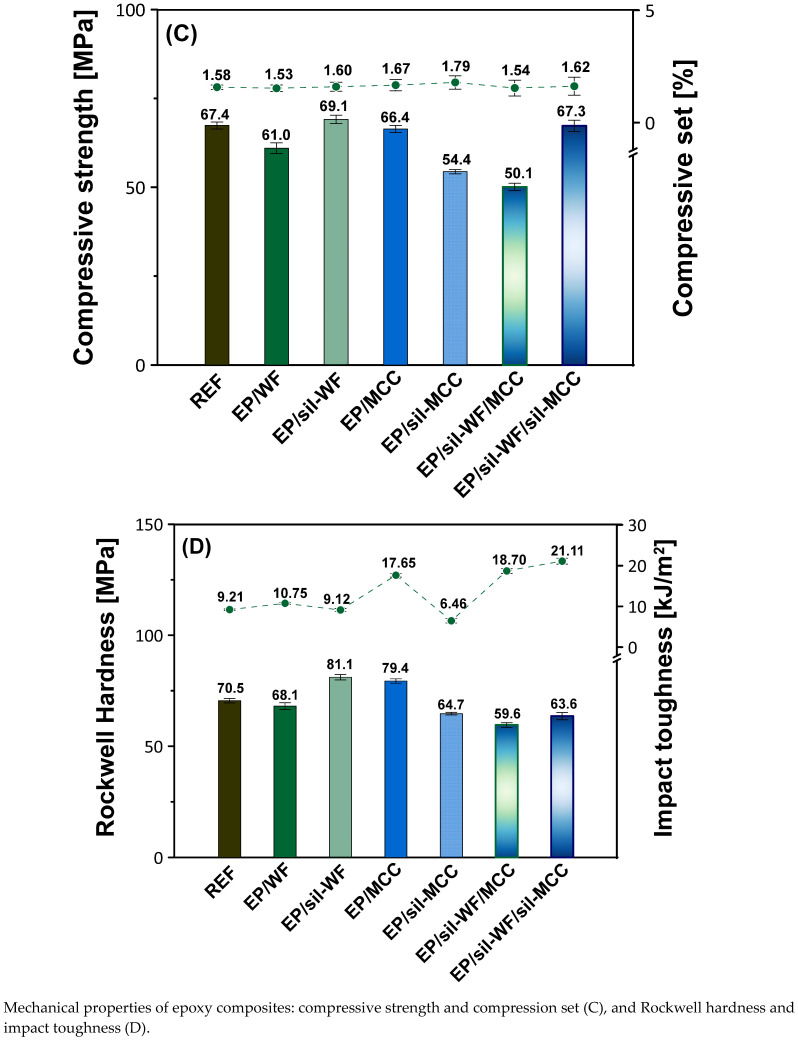
Mechanical properties of epoxy composites.

Based on the results of the mechanical tests (Figure 7A–C, mechanical properties of epoxy composites: tensile strength and elongation at break (A-1), modulus of elasticity (A-2), flexural strength and deflection (B-1), flexural elasticity modulus (B-2), compressive strength and compression set (C), and Rockwell hardness and impact toughness (D)), it was observed that the reference sample without cellulosic filler exhibited the highest tensile strength value (49.50 ± 1.11 MPa). The introduction of cellulosic particles resulted in a reduction in the tensile strength of the obtained materials by 6 to 36%. Among the compositions containing fillers, the highest tensile strength of 47.26 ± 3.15 MPa was recorded for the composition with unmodified microcrystalline cellulose (EP/MCC). Slightly lower values of tensile strength were observed for the compositions with the addition of modified fillers (43.66 ± 3.5 MPa for EP/sil-WF/sil-MCC, 42.84 ± 2.8 MPa for EP/sil-WF/MCC, and 42.34 ± 4.9 MPa for EP/sil-WF, respectively). At the same time, the composite containing MCC was characterized by the highest value of elongation at break (3.1 ± 0.5% compared with 3.02 ± 0.2% registered for REF). However, it is also worth highlighting that for composites containing two additives, EP/sil-WF/MCC and EP/sil-WF/sil-MCC, characterized by slightly lower tensile strength, the recorded values of elongation at break were comparable (2.7 ± 0.4% for EP/sil-WF/MCC and 2.6 ± 0.5% for EP/sil-WF/sil-MCC, respectively). A lower tensile strength of the composition with the addition of unmodified wood waste compared to the strength of the unfilled epoxy composition was also noted in studies conducted by Jain et al. [53], in which pineapple leaf fibers were used as the lignocellulosic filler in amounts of 5–25 wt.%. The reduction in tensile strength is probably caused by the incompatibility of the nature of the introduced filler fibers with the hydrophobic epoxy resin matrix, leading to poor adhesion between the composite components and the resulting uneven stress transfer. At the same time, in the case of waste wood flour, it was found that the silanization improved the compatibility of the filler with the epoxy matrix, resulting in an improvement in tensile strength by 25% compared to the composition with unmodified wood waste flour, EP/WF (31.45 ± 3.2 MPa–EP/WF and 42.34 ± 4.9 MPa–EP/sil-WF, respectively). On the other hand, in the case of MCC, the opposite effect of the modification was observed. The tensile strength value decreased by 21% compared to EP/MCC and by 25% compared to the reference sample without cellulosic filler (37.34 ± 2.7 MPa–EP/sil-MCC, 47.26 ± 6.4 MPa–EP/MCC, and 49.50 ± 1.1 MPa–EP, respectively). As shown in Figure 7, the Young’s modulus of the reference epoxy resin composition was recorded as 1141.5 ± 247.3 MPa. Introduction of WF in unmodified form resulted in a slight decrease in the value, while the addition of MCC in the epoxy composition increased the Young’s modulus to 1154.1 ± 125.2 MPa. Taking into account that a decrease in a material’s Young’s modulus indicates an increase in its elasticity and a decrease in its stiffness. In other words, it means that the material is more easily subjected to elastic deformation under the influence of force. The material with the addition of 5 wt.% of unmodified wood flour will be more susceptible to deformation than the composite containing 1 wt.% of MCC, as well as the reference material without the cellulose additive. The recorded values of the Young’s modulus for composites containing silanized filler particles (EP/sil-WF, EP/sil-MCC) indicate that the modification of the filler leads to obtaining materials characterized by greater flexibility in comparison to materials containing the additive in unmodified form.

A similar effect of the introduction of unmodified wood flour to the epoxy composition was noticed on the flexural strength of the material. In the case of EP/WF, the flexural strength decreased by 22% compared to the reference sample without the filler. However, after chemical modification, the bending strength of the material with this filler increased both in relation to the value recorded for the composite with unmodified wood flour and also in relation to the reference composition (61.97 ± 1.88 MPa–EP/sil-WF, 42.39 ± 0.49 MPa–EP/WF, and 54.55 ± 1.05 MPa–EP, respectively). In the case of the composite containing MCC, the flexural strength increased by 7.5%, but similarly to the results of the tensile strength test discussed before, the introduction of silanized MCC into the epoxy resin resulted in a deterioration of the flexural strength properties by, respectively, 18% comparing to the EP/MCC composition and 11% comparing to the EP composition. The introduction of two fillers in the form of sil-WF/MCC and sil-WF/sil-MCC in the amount of 5 wt.% resulted in improvement of the bending properties of the samples compared to the reference epoxy material (54.84 ± 1.44 MPa–EP/sil-WF/MCC and 58.43 ± 1.06 MPa–EP/sil-WF/sil-MCC compared to 54.55 ± 1.05 MPa–EP).

The trend of value distribution, described above, was also observed for compressive strength. The EP/sil-WF composite was characterized by the highest compressive strength of all tested samples. The recorded value was correspondingly larger by 2.5, 2.6, and 3.9% than the EP/sil_WF/sil-MCC, EP, and EP/MCC compositions, respectively. The introduction of a silanized additive in the epoxy composition resulted in obtaining a higher mechanical strength value than that recorded for compositions containing an unmodified cellulose filler. For the tested compositions, the compressive set was at a similar level of 1.52–1.79%. Additionally, the introduction of 5 wt.% of the additive to the epoxy composition most often resulted in a decrease in the Rockwell’s hardness of the final material by 3.4 to 15.5%. The exception was the composition containing 5 wt.% of silanized wood flour, for which an increase of approximately 10 MPa in the hardness value was noted (81.1 ± 0.7 MPa–EP/sil-WF and 70.5 ± 0.6 MPa–EP, respectively). Moreover, the addition of microcrystalline cellulose in an amount of 1 wt.% resulted in an increase in the hardness of the epoxy material compared to the reference sample (79.4 ± 1.3 MPa–EP/MCC, and 70.5 ± 0.6 MPa–EP, respectively). An increase in composite hardness associated with the addition of microcrystalline cellulose to epoxy resin was also observed in the studies of Liu et al. [54]. Generally, it was also observed that compositions containing cellulosic filler were characterized by larger values of impact toughness than the EP composition. This means that introducing an additive such as waste wood flour, microcrystalline cellulose, or both fillers resulted in materials with greater resistance to cracking under sudden, dynamic loads. A material with higher impact strength is more ductile and can absorb more impact energy before breaking. This means it is less susceptible to brittleness.

**Figure 8:**
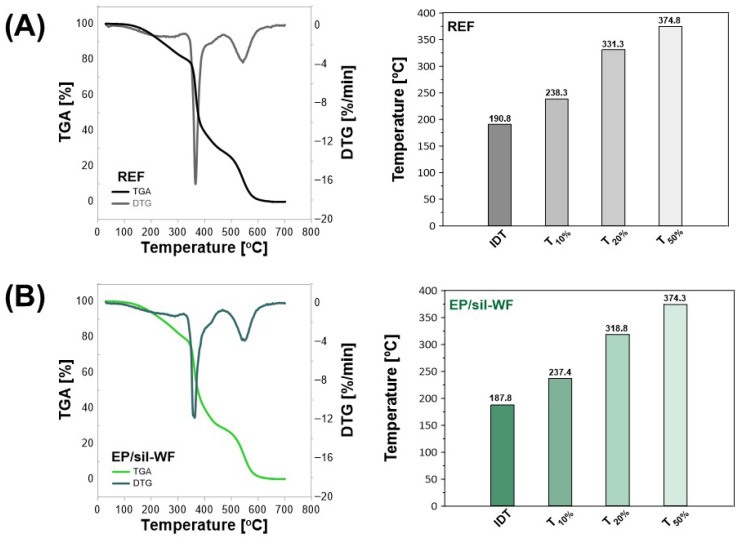

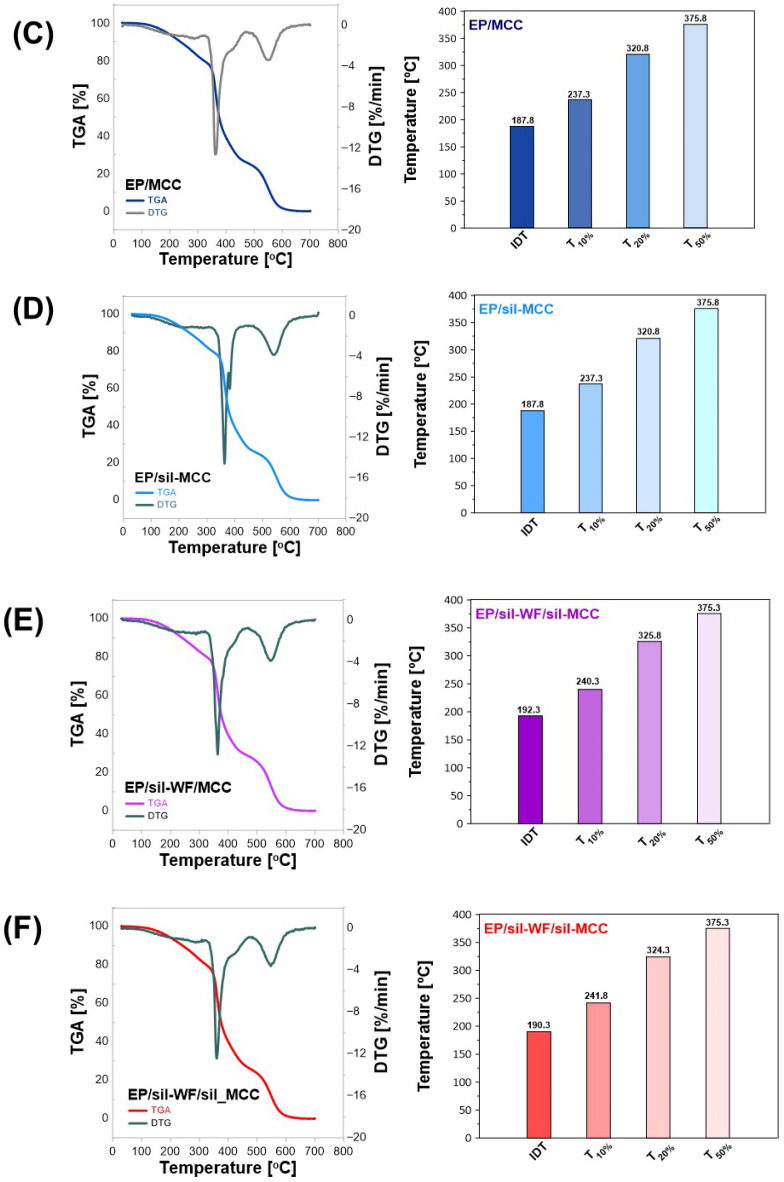
TGA and DTG analysis of crosslinked epoxy composites. Reference composition without filler (**A**); compositions with cellulosic fillers: silanized wood filler EP/sil-WF (**B**); microcrystalline cellulose EP/MCC (**C**); silanized microcellulose EP/sil-MCC (**D**), as well as epoxy composites with a combination of two additives; silanized wood filler with microcellulose EP/sil-WF/MCC (**E**); and silanized wood filler with silanized microcellulose EP/sil-WF/sil-MCC (**F**).

In the next stage of the research, the thermal stability of selected compositions was analyzed (Figure 8 and Table 2). For all analyzed cured epoxy compositions, the degradation temperatures were recorded, representing mass losses of 10, 20, and 50%, respectively. It is worth highlighting here that the selection of samples of epoxy materials for thermogravimetric analysis was based both on the choice of components for hierarchical composites and the obtained results of mechanical testing. Since generally the composite with unmodified wood flour was characterized by worse properties than the composite with silanized wood flour, only sil-WF was used to obtain compositions containing two fillers with a total additive content of 5% by weight (4 wt.% of silanized wood flour and 1 wt.% of unmodified or silanized microcellulose–EP/sil-WF/MCC and EP/sil-MD/sil-MCC compositions, respectively). An unfilled epoxy composition was the reference composition in the analysis of the thermal stability of epoxy composites presented below.

The first stage of the degradation of the tested samples was observed at a temperature range of 0–200 °C. The reduction in weight during that time is probably related to the removal of moisture present in the material. Then, the next stage might be assigned to a temperature range of 250–400 °C. During that time, the decomposition of hemicellulose/debonding of glycosidic linkages of the lignocellulosic fiber and the breakage of cellulose linkages are observed. Finally, in the last stage, in the temperature range of 500–800 °C, the decomposition of lignin and other aromatic rings with complex structures took place [55,56].

**Figure 9:**
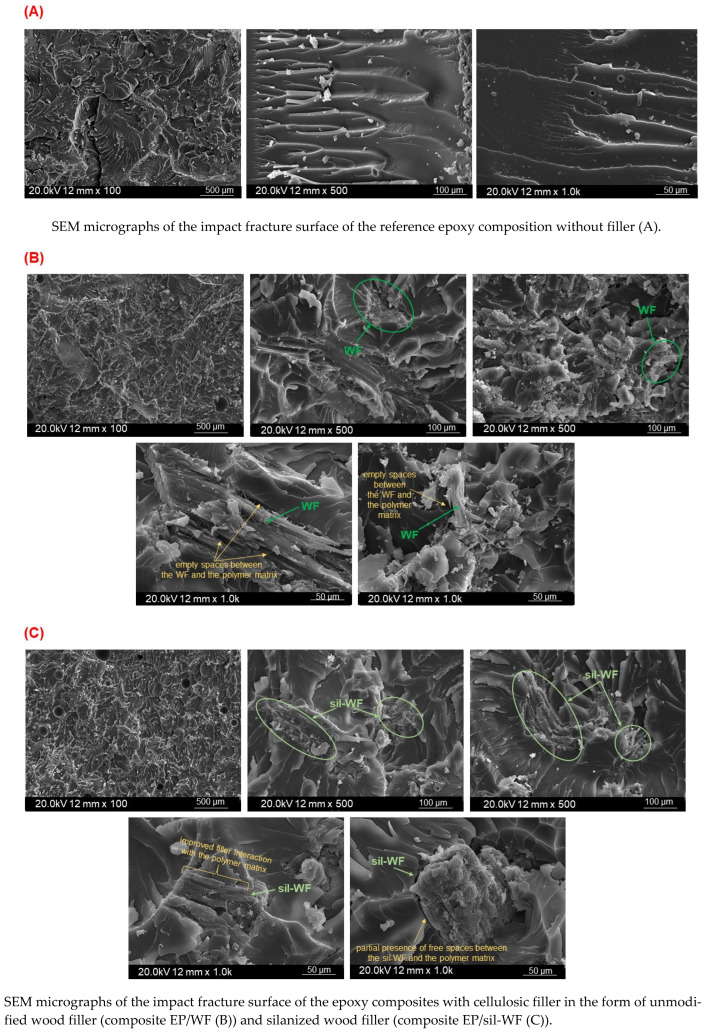

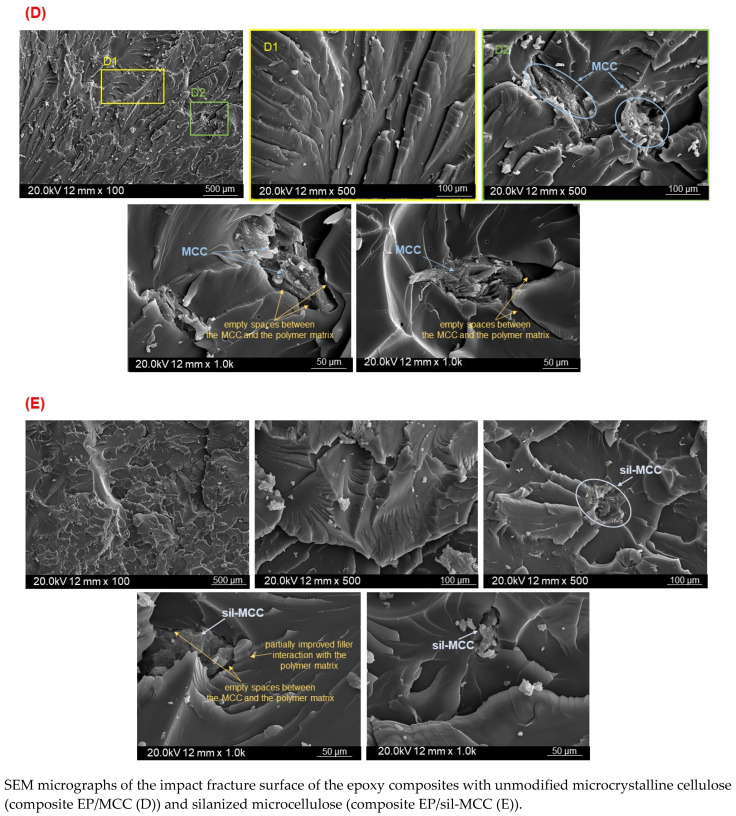

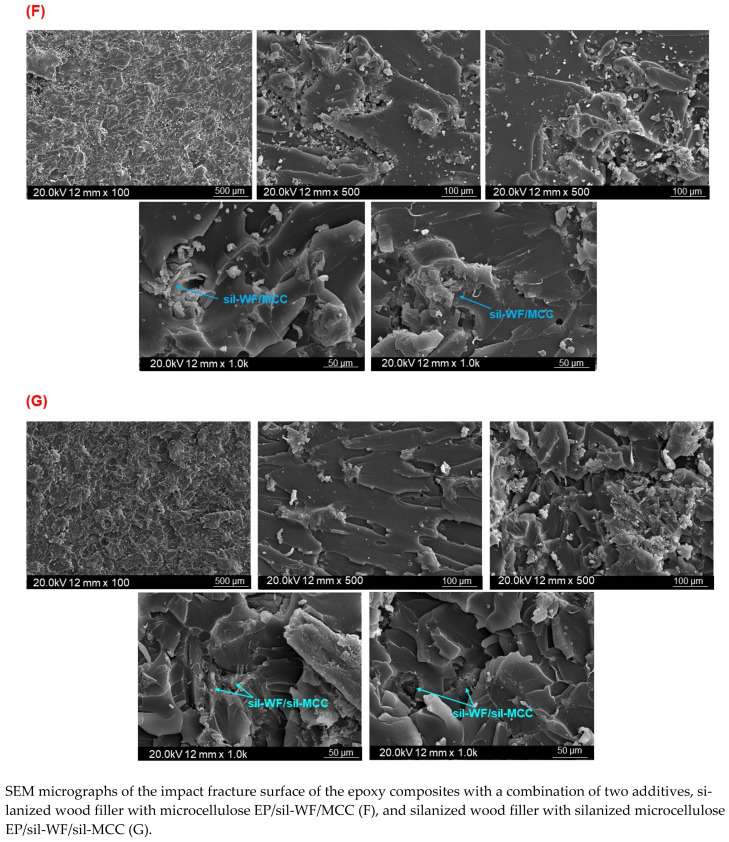
SEM micrographs of the impact fracture surface of the materials.

Comparing the thermal stability of composites with the addition of unmodified and silanized microcrystalline cellulose (Figure 8C,D), it was noted that the recorded temperature values associated with 10, 20, and 50% mass loss of the composite were higher for the sample EP/MCC containing the unmodified additive. The recorded difference for samples EP/MCC and EP/sil-MCC was the highest in the first stage, with T_10%_ for EP/MCC being larger by 5.5 °C, while for T_20%_, the difference was about 0.5 °C, and 3.5 °C for T_50%_, respectively. For the unfilled epoxy resin (Figure 9A), higher temperature values corresponding to a mass loss of 10% and 20% were recorded compared to those recorded for the MCC-filled composition (for EP—T_10%_ = 238.3 °C and T_20%_ = 331.3 °C, respectively, and for EP/MCC—T_10%_ = 237.3 °C and T_20%_ = 320.8 °C). However, the composite containing unmodified microcellulose and silanized wood flour was characterized by the highest IDT_2_ (IDT_2_EP/sil-WF/MCC_ = 192.8 °C) degradation temperature and temperature for a mass loss of 50% (T_50%_ = 375.8 °C), compared to the EP and EP/sMCC samples (IDT_2_EP/sil-WF/MCC_ = 190.3 °C, IDT_2_EP_ = 190.8 °C, respectively). In turn, the T_max_ value for the EP/sMCC composition is 1.5 °C higher than that for the composition containing unmodified microcellulose (T_max_EP/MCC_ = 361.3 °C and 362.8 °C for EP/sMCC, respectively). Furthermore, the composites with the addition of microcellulose were characterized by lower thermal stability compared to the EP sample. In turn, the highest value of solid residue after the degradation process was recorded at 1.5% for the composition with unmodified microcellulose. For the reference sample, the solid residue was approximately 1.2%, and for the composition with sil-MCC, 1.0%. The composite with the addition of silanized wood waste (EP/sil-WF, Figure 8B) was characterized by lower values of characteristic mass-loss temperatures, T_10%_ = 237.4 °C, T_20%_ (318.8 °C), and T_50%_ (374.3 °C), compared to the degradation temperatures recorded for the reference EP composition. The T_max_ temperature for EP/sil-WF was 360.8 °C, which is also lower than the T_max_ temperature recorded for the reference unfilled epoxy composition. A lower T_max_ temperature value for the composition with added lignocellulosic fiber (EP/bamboo fiber 5 wt.%) was also observed by Wang et al. [35]. By conducting a thermal analysis of the composition with the addition of two different fillers (Figure 8E,F), an improvement in thermal stability was observed compared to the reference sample (EP, Figure 8A). The degradation temperatures T_10%_, T_20%_, and T_50%_ for the EP/sil-WF/MCC composition were 240.3 °C, 325.8 °C, and 375.8 °C, respectively, while for the EP/sil-WF/sil-MCC composition, T_10%_ = 241.8 °C; T_20%_ = 324.3 °C; and T_50%_ = 375.3 °C. The sample containing two modified fillers (EP/sil-WF/sil-MCC) had a T_max_ of 371.8 °C, while for the sample filled with silanized wood flour and unmodified MCC, the T_max_ was 10 degrees lower (T_max_ = 361.3 °C).

In the following step of the research, the obtained materials were subjected to the morphology analysis, conducted by the SEM method, using their surface of impact fracture (Figure 9—*Figure 9A presents SEM micrographs of the impact fracture surface of the epoxy materials of reference composition without filler.* *Figure 9B–G—composites with cellulosic filler in the form of unmodified wood filler–composite EP/WF (B); silanized wood filler–composite EP/sil-WF (C); microcrystalline cellulose–composite EP/MCC (D); silanized microcellulose–composite EP/sil-MCC (E); and epoxy composites with a combination of two additives: silanized wood filler with microcellulose EP/sil-WF/MCC (F); and silanized wood filler with silanized microcellulose EP/sil-WF/sil-MCC (G)*). On the surface of the reference sample without the cellulosic filler (Figure 9A), several brittle fractures are present. In turn, in Figure 9B,C, microphotographs of the surface fracture of a composite containing unmodified or silanized wood flour are shown. By analyzing the entire fracture surface and assuming that the compositions were prepared in the same manner, it can be concluded that the chemical modification may have had a significant impact on the distribution and the interaction of the lignocellulosic additive within the polymer matrix. In the case of compositions with unmodified wood flour (Figure 9B), voids are visible in the areas of direct contact between the filler and the epoxy matrix. This indicates that the polymer matrix did not completely coat the filler grains. However, when it comes to EP/sil-WF (Figure 9C), the improved interaction of the modified wood flour with the polymer matrix is visible. This correlates with the improved mechanical properties of the EP/sil-WF composition compared to EP/WF.

A different situation was recorded for microcrystalline cellulose. In this case, as a result of the filler modification, a deterioration in the distribution and interaction between the additive and the polymer matrix was observed. Hence, a significant deterioration in the mechanical properties of the EP/sil-MCC composition was observed compared to EP/MCC. This phenomenon can be explained by the observed and earlier-discussed (Section 2.1) morphological changes in the filler itself. Due to the modification, an enlargement of particles of microcrystalline cellulose was observed. The silanized microcellulose tended to agglomerate, creating much larger clusters with sizes of approximately 200–550 µm (Figure 5). Meanwhile, the dimensions of particles of unmodified MCC were within the range 60–200 µm. In the cluster form, it was much more difficult to observe any possible increase in surface roughness compared to the unmodified MCC, which could lead to improvement in the interaction with the polymer matrix. However, even assuming that such roughness was formed, it more likely contributed to the increased interaction of individual grains and the formation of larger and compact clusters, which were difficult to separate and distribute evenly in the polymer matrix in the process of combining the filler with the polymer composition. On the SEM microphotographs (Figure 9D,E), significant empty spaces are clearly visible between the filler grains and the polymer matrix. In the case of sil-MCC, these empty spaces stand out to a lesser extent, but only locally, and unfortunately, do not cover the entire filler surface.

As would be expected, in the SEM micrographs for the composition EP/sil-WF/MCC and EP/sil-WF/sil-MCC, the interface between the polymer matrix and filler is clearly noticeable. Also, in the case of these compositions, pullouts of filler particles from the polymer matrix are visible. However, fillers, revealed within the microphotographs, are coated with a polymer matrix in most of their area. In this case, the mechanical properties and thermal stability are primarily related to the characteristics of the additives themselves and their mutual influence. Based on the recorded SEM micrographs, it is not possible to clearly determine the influence or interaction of fillers in the polymer matrix. It can only be stated that despite the procedures applied, such as washing and blowing the samples for SEM analysis before their final sputtering with a gold layer, a significant number of fine-grained fragments are visible on the surface of the EP/sil-WF/MCC composite. In the case of EP/sil-WF/sil-MCC, these unbound fragments are much larger and less numerous.

## 3. Materials and Methods

Materials. The oak wood waste flour (WF, wood flour particle dimensions < 0.04 mm; the material was obtained as waste during the production of parquet flooring from FHU Parkiety Smolik, Lanckorona, Poland), microcrystalline cellulose (microcellulose, MCC, Avicel PH-101, CAS: 9004-34-6, is a loose, white, and odorless powder with an average diameter of 8 μm, as supplied by the manufacturer Sigma-Aldrich, Poznan, Poland), UNISILAN U-13 (3-aminopropyltriethoxysilan, C_9_H_23_NO_3_Si, CAS: 919-30-2, is a clear, slightly yellow liquid: assey–99% and density–0.951 g/cm^3^ at 25 °C UniSil Sp. z.o.o., Tarnow, Poland), Epidian 6 (EP6, low-molecular-weight bisphenol A epoxy resin, EV = 0.536 mol/100 g, general CAS number for BPA-based epoxy resins: 25068-38-6, CIECH Sarzyna S.A. Nowa Sarzyna, Poland), and IDA hardener (an amine hardener based on isophorone diamine for liquid epoxy resins, CAS: 38294-64-3, CIECH Sarzyna S.A.Nowa Sarzyna, Poland), BYK A530 deaerator (BYK Additives, Wesel, Germany).

### 3.1. Preparation of Filler

#### 3.1.1. Waste Wood Filler

**Figure 10:**
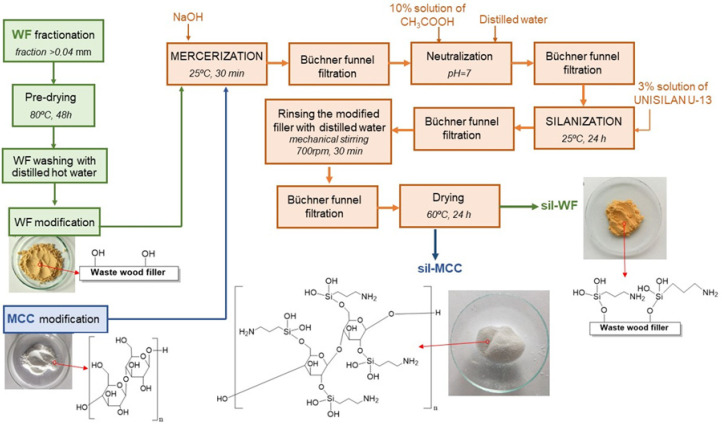
Scheme of the chemical modification of fillers for epoxy composites.

Separated into individual fractions and pre-dried for 48 h at 80 °C, waste oak flour was subjected to chemical modification (Figure 10) and then used as a filler for epoxy composites. In total, 150 g of the wood waste was placed in a beaker, and 1000 mL of 10% sodium hydroxide solution was added. The resulting suspension was stirred for 30 min using a mechanical stirrer (IKA RW 20 digital, IKA Poland, Krakow, Poland). After this time, it was filtered under reduced pressure using a Buchner funnel (Chemland, Stargard, Poland). Then the modified flour was washed on the filter with portions of a 10% acetic acid solution. Washing of the mercerized WF with acetic acid was continued until the filtrate reached neutral pH. The wet, mercerized wood waste was then silanized using a 3% solution of 3-aminopropyltriethoxysilane in a mixture of methanol and water (1:1). A solution with a pH of 4.0 was used. The desired pH of the silane was achieved by adding acetic acid in portions (10 cm^3^ each). The wood flour was treated with silane at room temperature for 20 h. The modified waste wood was then filtered on a Buchner funnel and washed with 500 mL of distilled water. Finally, the waste was dried for 24 h at 60 °C. The silanized wood flour (sil-WF) was stored in a desiccator.

#### 3.1.2. Microcrystalline Cellulose

Ten grams of microcrystalline cellulose was placed in a 200 mL beaker, and 100 mL of a 10% sodium hydroxide solution was added (Figure 10). Such a suspension was stirred for 30 min using a mechanical stirrer. The mercerized microcellulose was then filtered using a Buchner funnel and washed with two portions of 10% acetic acid solution. The wet, mercerized microcellulose was treated with a 3% solution of 3-aminopropyltriethoxysilane in a methanol/water mixture (1:1). The silane solution’s pH of 4.0 was adjusted by adding to the silane solution two portions (10 cm^3^ each) of acetic acid. The silanization of the microcellulose was carried out at room temperature for 20 h. The modified microcellulose was then washed with 50 mL of distilled water on a Buchner funnel. The silanized microcrystalline cellulose (sil-MCC) was dried for 24 h at 60 °C.

### 3.2. Preparation of Epoxy-Waste Wood Composites

Seven epoxy compositions were obtained, including a reference composition without a filler, as well as systems with the addition of modified or unmodified wood flour, modified/unmodified microcellulose, and composites with two additives (Table 3).

After weighing the appropriate amount of epoxy resin, in the case of filled compositions, wood flour (either chemically modified or unmodified) was added at a 5% by weight concentration and/or microcellulose at a 1% by weight concentration. In the case of compositions containing two fillers, the total additive content was 5 wt.%, including, respectively, 4 wt.% of wood flour and 1 wt.% of microcellulose. The mixture was then mixed intensively using a mechanical mixer. Mixing was carried out for 30 min at 700 rpm. Next, a calculated and weighed amount of hardener ($m_{hardener}=EV\cdot 95$, where $m_{hardener}$—amount [g] of IDA amine hardener to crosslink 100 g of epoxy resin, and EV—epoxy value [mol/100 g epoxy resin], where an amount of epoxy functional groups in Epidian 6 epoxy resin) and BYK A530 deaerator at 1% by weight of the entire composition was added to the homogeneous mixture. The resulting epoxy system was again mixed using a mechanical stirrer (5 min at 1000 rpm) and then subjected to deaeration for 3 min at a pressure of 0.8 MPa. The deaerated mixture was poured into Teflon molds. Curing lasted 24 h and was carried out at room temperature. Then, the obtained samples for mechanical tests were removed from the molds, seasoned for 7 days, and post-cured at 80 °C for 24 h.

### 3.3. Characterization of Composite Materials

#### 3.3.1. Spectroscopic Analysis (ATR/FT-IR)

The analysis of the chemical structure of fillers was performed using the Fourier Transform Infrared Spectroscopic method (FT-IR) with the Thermo Scientific Nicolet iS5 spectrometer equipped with a single reflection diamond crystal. FTIR analysis was conducted in a spectral range of 400–4000 cm^−1^ (number of scans: 16, resolution: 4000, apodization: N-B strong, phase correction: Mertz, number of background scans: 16, background enhancement: 1.0, and using a DTGS KBr detector, Thermo Electron Corporation, Waltham, MA, USA). The measurements were carried out at room temperature. A small amount of filler sample was placed onto the crystal attenuated total reflectance unit (ATR), manufactured by Thermo Electron Corporation (Waltham, MA, USA) and pressed down with a screw to compress the analyzed sample. The spectra were presented as a function of transmittance T [%] from the wavenumber v [cm^−1^] in the range of 600–4000 cm^−1^.

#### 3.3.2. Wide Angle X-Ray Diffraction (WAXD)

The X-ray diffraction investigations were performed using the D2 PHASER apparatus from Bruker (Billerica, MA, USA) in the Bragg–Brentano configuration with the following parameters: the range of the 2θ angle was 3–40°, the size of the slit on the lamp was 0.5 mm, and the aperture was 1 mm. The counting time was 0.3°/min with a step of 0.020°. Samples were in powder form.

#### 3.3.3. The Mechanical Properties of Epoxy Composites

The mechanical properties of the prepared epoxy composites reinforced with cellulosic filler were tested according to previously described procedures [39], including determination of tensile (PN-EN ISO 527-1:2012 [57]), flexural (PN-EN ISO 178:2011 [58]), compressive (PN-EN ISO 604:2006 [59]), and Charpy impact (PN-EN ISO 1792:2001 [60]) properties. Tests were performed using samples of epoxy composites in the shape of paddles, beams, and rollers. Each of the tests of individual mechanical properties was carried out on a set of 5 samples; the obtained results were averaged, and the measurement error was determined for each test. The tensile strength, elongation at break, modulus of elasticity, flexural strength, elasticity, flexural modulus, deflection, compressive strength, and compression set were tested on the Zwick 1445 apparatus (Poland), while determination of impact strength without notching by the Charpy’s method was tested using the ZORN PSW 4J Digital apparatus (ZORN instruments, Stendal, Germany).

- The determination of tensile properties, including the tensile strength, elongation at break, and modulus elasticity, was determined according to PN-EN ISO 527-1:2012 standard on samples in the form of paddles (type B) [dimensions of measurement section: 4 × 10 mm and length of 50 mm] and using the Zwick apparatus equipped with an extensometer. Applied testing speed of measurements: 5 mm/min.
- The determination of flexural properties, including flexural strength, elasticity, flexural modulus, and deflection, was tested according to PN-EN ISO 178:2011 standard on samples in the form of a cuboid beam with the cross-section dimensions of 4 × 10 mm, 64 mm spacing between supports, and a testing speed of 10 mm/min.
- The determination of compressive strength and compression set was tested according to PN-EN ISO 604:2006 standard on rollers with 10 mm diameter and 25 mm height, with an applied testing speed of 0.8 mm/min.
- The determination of impact strength without notching by the Charpy’s method was tested based on PN-EN ISO 1792:2001 standard using the ZORN PSW 4J Digital apparatus and cuboid beams with the cross-section of dimensions 4 × 10 mm.

#### 3.3.4. Morphological Analysis

Morphological analysis was performed with a JEOL JSM 6010LA scanning electron microscope (JOEL Ltd., Tokyo, Japan), and micro images of the impact-fractured surfaces of the epoxy composites and pre-dried filler particles were recorded. The images recorded using an optical microscope were taken at approximately 4×. SEM micrographs were recorded using samples coated with a thin film of gold at an acceleration voltage of 5 kV. In the case of all analyzed additives, the comparison of the grain sizes and morphological changes was made upon the detailed observation of the entire sample subjected to the SEM analysis, and the selection of five of the most representative microimages for each additive. From the recorded five different detailed micrographs, one was selected to illustrate the differences between additives, which were presented in Figure 4 and Figure 5.

#### 3.3.5. Thermogravimetric Analysis

The thermogravimetric analysis was performed using a Netzsch TG 209 F1 Libra thermal analyzer (Netzsch, Selb, Germany). The samples with mass approx. 5 mg were heated in an open corundum pan from 30 °C up to 600 °C at a heating rate of 10 °C min^−1^ under an inert atmosphere.

## 4. Conclusions

The main purpose of the presented research was the synthesis and characterization of various epoxy composites with green additives in the form of waste oak flour and microcrystalline cellulose. The applied fillers were used in unmodified and silanized form. Chemical modification was performed to reduce the hydrophilic properties of cellulosic fillers. Based on previous studies, two modification methods (mercerization and silanization) were combined to reduce the influence of hydroxyl groups associated with cellulosic compounds, purify the additives from additional substances or other impurities deposited during the production process, and increase compatibility between the additive and polymer matrix. Increased filler/polymer adhesion resulted from the combined effects of bridge-like silane and the introduction of fillers with different spatial architectures. The addition of filler in the form of unmodified wood flour (WF) or microcellulose (MCC) resulted in materials with reduced tensile strength and enhanced impact fracture compared to the unfilled composition. Additionally, the composite with MCC was characterized by increased bending strength. The performed chemical modification had the greatest impact on increasing the interaction of the waste wood flour with the epoxy matrix. As a result, a greater degree of adhesion of the additive to the matrix was observed on recorded microimages, resulting in improved mechanical properties. Composite EP/sil-WF was characterized by improved flexural and compressive properties compared to both EP/WF and unfilled reference composition. In turn, silanization of microcellulose resulted in deterioration of the mechanical properties of the obtained composites compared to the results recorded for materials with unmodified MCC. The observed properties could be caused mainly by the spatial structure of sil-MCC, which tended to agglomerate, creating much larger, compact, and difficult-to-separate clusters within the polymer matrix. Simultaneously, the mechanical properties and thermal stability of hierarchical epoxy composites containing two fillers, sil-WF and MCC (EP/sil-WF/MCC composition) or sil-WF and sil-MCC (EP/sil-WF/sil-MCC), primarily result from the characteristics of the additives themselves and their mutual influence. The synergistic effect of the two applied fillers was most evident in the increase in impact toughness and thermal stability related to IDT_2_ and T_50%_. The composites EP/sil-WF/MCC and EP/sil-WF/sil-MCC were more resistant by approximately 1–11 MPa to sudden cracking under the dynamic loads and approx. by 1–3 °C to thermal degradation than the composites containing one of the selected cellulosic additives or the reference composition without fillers.

## Figures and Tables

**Table 1:** FT-IR analysis of WF, sil-WF, MCC, and sil-MCC [41,42,43,44].

Bands				Assigned Vibrations
MCC	sil-MCC	WF	sil-WF	
3637–3000	3629–2991	3671–3000	3629–2999	Stretching vibrations in OH groups and water (3629–3000 cm^−1^, 3600–3750 cm^−1^ free OH groups, respectively)
3000–2844	3000–2819	2985–2822	3000–2828	Stretching vibrations in CH_2_, CH, and CH_3_ (2822–3000 cm^−1^)
1743	-	1732	-	Stretching vibrations in -C=O of hemicellulose or lignin
1642	1638	1600	1597	Stretching vibrations in -C=C- (1597–1600 cm^−1^, including benzene ring, possibly from lignin)
1437	1434	1422	1459	1459 cm^−1^ bending vibrations in -CH_2_, while 1422–1437 cm^−1^ bending vibrations in -CH_2_ and -OCH
1366	1369	1366	1422	Scissor -CH_2_, flat deformation -C-H
1314	1316	1318	1321	Bending vibrations in -COH and -CCH (1314–1321 cm^−1^)
1202	1159	1228	1227	Bending vibrations in -CCH and -COH, as well as wagging vibrations in -CH_2_ (1202–1228 cm^−1^)
1161	1161	1164	1157	Predominantly stretching vibrations in -C-O bonds (mainly v_as_(C-O-C) in bridge structure), 1157–1161 cm^−1^
-	1080	-	1054	Stretching vibrations in Si-O (1080–1054 cm^−1^)
1029	1029	1035	1032	Stretching vibrations in -C-O bonds (1029–1035 cm^−1^)
900	895	903	897	Non-planar deformation -C-H; β-glycosidic bonds between monosaccharides

**Table 2:** Thermal stability of epoxy compositions with cellulosic fillers.

	Composition					
	EP	EP/sil-WF	EP/MCC	EP/sil-MCC	EP/sil-WF/MCC	EP/sil-WF/sil-MCC
**Char residue [%]**	1.2	1.6	1.5	1.0	1.7	1.5

**Table 3:** Composition based on low-molecular-weight epoxy resin EPIDIAN 6.

Sample	Polymer Matrix	Hardener	Deaerator	Type and Amount of Filler [wt.%]			
				WF	sil-WF	MCC	sil-MCC
EP	Epidian 6	Isoforone dimane	BYK A530	–	–	–	–
EP/WF				5	–	–	–
EP/sil-WF				–	5	–	–
EP/MCC				–	–	1	–
EP/sil-MCC				–	–	–	1
EP/sil-WF/MCC				–	4	1	–
EP/sil-WF/sil-MCC				–	4	–	1

## Data Availability

The data presented in this study are available on request from the corresponding author.

## Abbreviations

The following abbreviations are used in this manuscript:

EP	Cured epoxy composition based on low-molecular-weight epoxy resin Epidian 6.
EP/WF	Cured epoxy composite based on low-molecular-weight epoxy resin Epidian 6 filled with 5 wt.% of unmodified wood flour.
EP/sil-WF	Cured epoxy composite based on low-molecular-weight epoxy resin Epidian 6 filled with 5 wt.% of silanized wood flour.
EP/MCC	Cured epoxy composite based on low-molecular-weight epoxy resin Epidian 6 filled with 1 wt.% of unmodified microcrystalline cellulose.
EP/sil-MCC	Cured epoxy composite based on low-molecular-weight epoxy resin Epidian 6 filled with 1 wt.% of silanized microcrystalline cellulose.
EP/sil-WF/MCC	Cured epoxy composite based on low-molecular-weight epoxy resin Epidian 6 filled with 4 wt.% of silanized wood flour and 1 wt.% of unmodified microcrystalline cellulose.
EP/sil-WF/sil-MCC	Cured epoxy composite based on low-molecular-weight epoxy resin Epidian 6 filled with 4 wt.% of silanized wood flour and 1 wt.% of silanized microcrystalline cellulose.
